# Serologic markers of Epstein-Barr virus reactivation are associated with increased disease activity, inflammation, and interferon pathway activation in patients with systemic lupus erythematosus

**DOI:** 10.1016/j.jtauto.2021.100117

**Published:** 2021-08-31

**Authors:** Rebecca A. Wood, Lauren Guthridge, Emma Thurmond, Carla J. Guthridge, Joseph M. Kheir, Rebecka L. Bourn, Catriona A. Wagner, Hua Chen, Wade DeJager, Susan R. Macwana, Stan Kamp, Rufei Lu, Cristina Arriens, Eliza F. Chakravarty, Aikaterini Thanou, Joan T. Merrill, Joel M. Guthridge, Judith A. James

**Affiliations:** a Arthritis and Clinical Immunology Program, Oklahoma Medical Research Foundation, Oklahoma City, OK, 73104, USA; b Departments of Medicine and Pathology, University of Oklahoma Health Sciences Center, Oklahoma City, OK, 73104, USA

**Keywords:** Systemic lupus erythematosus, Epstein-barr virus, Interferon, Antibodies, Disease activity

## Abstract

SLE is a clinically heterogeneous disease characterized by an unpredictable relapsing-remitting disease course. Although the etiology and mechanisms of SLE flares remain elusive, Epstein-Barr virus (EBV) reactivation is implicated in SLE pathogenesis. This study examined the relationships between serological measures of EBV reactivation, disease activity, and interferon (IFN)-associated immune pathways in SLE patients. Sera from adult SLE patients (n = 175) and matched unaffected controls (n = 47) were collected and tested for antibodies against EBV-viral capsid antigen (EBV-VCA; IgG and IgA), EBV-early antigen (EBV-EA; IgG), cytomegalovirus (CMV; IgG), and herpes simplex virus (HSV-1; IgG). Serological evidence of EBV reactivation was more common in SLE patients compared to controls as demonstrated by seropositivity to EBV-EA IgG (39% vs 13%; p = 0.0011) and EBV-VCA IgA (37% vs 17%; p = 0.018). EBV-VCA, CMV1, and HSV-1 IgG seropositivity rates did not differ between SLE patients and controls. Furthermore, concentrations of EBV-VCA (IgG and IgA) and EBV-EA (IgG) were higher in SLE patients. SLE patients with high disease activity had increased concentrations of EBV-VCA IgA (mean ISR 1.34 vs. 0.97; p = 0.041) and EBV-EA IgG levels (mean ISR 1.38 vs. 0.90; p = 0.007) compared with those with lower disease activity. EBV reactivation was associated with enhanced levels of the IFN-associated molecule IP-10 (p < 0.001) and the soluble mediators BLyS (p < 0.001) and IL-10 (p = 0.0011). In addition, EBV-EA IgG responses were enriched in two previously defined patient clusters with robust expression of IFN and inflammatory or lymphoid and monocyte responses. Patients in these clusters were also more likely to have major organ involvement, such as renal disease. This study supports a possible role for EBV reactivation in SLE disease activity.

## Highlights

• Serologic markers of EBV reactivation are more common in SLE patients.
• Elevated EBV reactivation is associated with higher SLE disease activity.
• EBV serologic reactivation correlates with elevated IP-10, IL-10, and BLyS levels.
• EBV reactivation occurs in SLE clusters with robust inflammatory and IFN responses.

## 1 Introduction

Systemic lupus erythematosus (SLE) is a chronic progressive autoimmune disease with profound clinical heterogeneity, multiorgan inflammation, complex pathogenesis, and a relapsing-remitting course. SLE flares are characterized by the development and progressive accrual of autoantibodies, exaggerated pro-inflammatory type I interferon (IFN) production, and impaired apoptotic clearance, which drives cumulative damage to tissues and organs, such as the skin, joints, and kidneys. Despite decades of research, the molecular mechanisms and etiology of SLE are not completely understood. SLE is concordant in 34% of monozygotic twins compared to 3% of dizygotic twins [1,2], and 10–12% of SLE patients have a first or second degree relative with SLE compared to <1% of control participants [3,4], indicating a genetic component. However, genetic discordance in these studies highlights the complex interplay between genetic risk and environmental exposures in SLE pathogenesis.

Epstein-Barr virus (EBV) is a common herpesvirus implicated in several carcinomas [5], lymphoproliferative conditions [6,7], and autoimmune diseases [[8], [9], [10], [11]]. Despite near-ubiquitous exposure in adult populations worldwide, EBV exposure is more common in pediatric and adult SLE patients compared to unaffected controls [10,12], suggesting that EBV infection may influence SLE pathogenesis in genetically predisposed individuals. EBV establishes life-long latency in B cells and expresses a limited number of genes. EBV occasionally reactivates the lytic cycle, resulting in the expression of lytic antigens, such as viral capsid antigen (VCA) and early antigen (EA). Initial EBV infection induces antibody responses to EBV viral capsid antigen (EBV-VCA) and EBV early antigen (EBV-EA) [13]. During latency, EBV-VCA IgG antibodies persist at lower levels, while EBV-EA IgG and EBV-VCA IgA antibodies are not detectable [13]. EBV reactivation increases EBV-VCA IgG antibodies and induces detectable EBV-VCA IgA and EBV-EA IgG [13]. Therefore, serology can distinguish individuals with latent infection or viral reactivation.

Previous studies demonstrated higher EBV viral loads in peripheral blood mononuclear cells [11,14], enhanced seroprevalence of EBV-EA IgG antibodies [[15], [16], [17], [18], [19]], and increased expression of lytic genes [14,20] in SLE patients compared with healthy controls. Together, these studies suggest that SLE patients have more frequent EBV reactivation. Furthermore, serological markers of EBV reactivation are associated with transitioning to SLE [21], suggesting that EBV reactivation may play a role in the development and progression of SLE. There are several proposed mechanisms for how EBV contributes to SLE pathogenesis. The EBV genome encodes human homolog proteins, including the latent protein, EBV nuclear antigen-1 (EBNA-1), and the lytic protein, viral IL-10 (vIL-10), that alter humoral and inflammatory immune responses in SLE [[22], [23], [24]]. Several EBNA-1 epitopes cross-react with SLE autoantigens, including Sm and Ro [22,25,26], and EBV reactivation is associated with a higher prevalence of several SLE-associated autoantibodies [19,21,27,28]. In addition, EBV induces type I IFN production by plasmacytoid dendritic cells [29,30], enhancing systemic inflammation during SLE flares. However, the effects of EBV reactivation on IFN-associated responses in SLE patients are still unclear.

In this study, we compared serological measures of EBV reactivation in SLE patients compared to unaffected controls. We stratified SLE patients based on disease activity to evaluate the associations between EBV reactivation and disease flare and determined whether EBV reactivation correlates with elevated inflammatory and IFN-associated responses. Our results provide further evidence that EBV reactivation influences SLE pathogenesis.

## 2 Materials and methods

### 2.1 Study participants

This study was approved by the Institutional Review Board of the Oklahoma Medical Research Foundation and used samples obtained previously from the Oklahoma Cohort for Rheumatic Diseases [31]. Adult SLE patients (n = 198) and matched unaffected controls (n = 47) were recruited through the Oklahoma Rheumatic Disease Research Cores Center and provided informed consent at the time of the initial blood draw. Blood samples and clinical and demographic information were collected as previously described [31] (Table 1). SLE patients met the American College of Rheumatology (ACR) SLE classification criteria (ACR ≥4) [32]. Disease activity was assessed using the SELENA-modified SLEDAI [33], and SLEDAI scores ≥6 and < 4 were considered high and low disease activity, respectively.

**Table 1:** Study participant demographics.

Group	SLE High (SLEDAI ≥6)	SLE Low (SLEDAI <4)	Control
Total, N^a^	93	82	47
Age in years, mean	39	47	43
Female, n (%)	83 (89)	73 (89)	45 (95)
Race, n (%)			
European American	38 (41)	45 (55)	24 (51)
African American	25 (27)	23 (28)	17 (36)
Asian	14 (15)	6 (7)	6 (13)
Mixed-race	16 (17)**	8 (10)	0 (0)

**P-value < 0.01 vs. controls. SLE, systemic lupus erythematosus; SLEDAI, systemic lupus erythematosus disease activity index.

a Total number of samples from 175 SLE patients and 47 controls. Statistical significance was determined using a two-tailed student's t-test or z-score test of population proportions.

### 2.2 Anti-viral antibody responses

Antibodies against EBV-VCA (IgG and IgA), EBV-EA (IgG), cytomegalovirus (CMV, IgG), and herpes simplex virus-1 (HSV-1, IgG) were measured using commercial ELISAs (Wampole Laboratories) as previously described [19]. Results are presented as units of the international standardized ratio (ISR). An ISR >1.1 was considered positive, and an ISR <0.9 was considered negative [19]. Equivocal results (ISR 0.91–1.09) were re-tested, and repeat equivocal results were dropped from the analysis.

### 2.3 Soluble mediators

IFN-induced protein 10 (IP-10) and IL-10 plasma levels were analyzed using xMAP multiplex assays (Affymetrix), and B lymphocyte stimulator (BLyS) plasma levels were assessed by ELISA (R&D Systems) as previously described [34]. As our previous study demonstrated limited non-specific binding with SLE and control samples [34], a rheumatoid factor blocking agent was not used in our Luminex based assays. Quality control was performed for inter- and intra-assay validity [34].

### 2.4 Data analysis

Samples with missing serological data and patients with SLEDAI scores of 4 or 5 were excluded from all analyses, resulting in a total of 222 samples from 175 SLE patients. Quantitative variables were compared by a two-tailed student's t-test and categorical variables by a two-tailed z-score test of population proportions. For SLE patients with two time-points (n = 105), 52 samples were selected at random for high disease activity and 53 samples for low disease activity. A resampled student's t-test with 1000 permutations was used to compare serological measures in SLE patients with high and low disease activity. Clustering was performed using random forest analysis as previously described [31]. R version 3.6.3 was used for all analyses, and P values less than 0.05 were considered statistically significant.

## 3 Results

### 3.1 Patient characteristics

We collected plasma from 175 SLE patients experiencing low (SLEDAI <4) or high (SLEDAI ≥6) disease activity and 47 demographically matched unaffected controls to determine the association between SLE disease activity and EBV reactivation. Of the SLE patients, 105 provided samples from both high and low disease activity time points (mean time difference = 1.7 years). Demographics of the study participants are presented in Table 1. No significant differences were observed in average age or sex between SLE patients or controls. However, a significantly higher proportion of SLE patients with high disease activity were mixed-race compared with controls.

### 3.2 SLE patients exhibit more frequent EBV reactivation compared to healthy controls

**Fig. 1:**
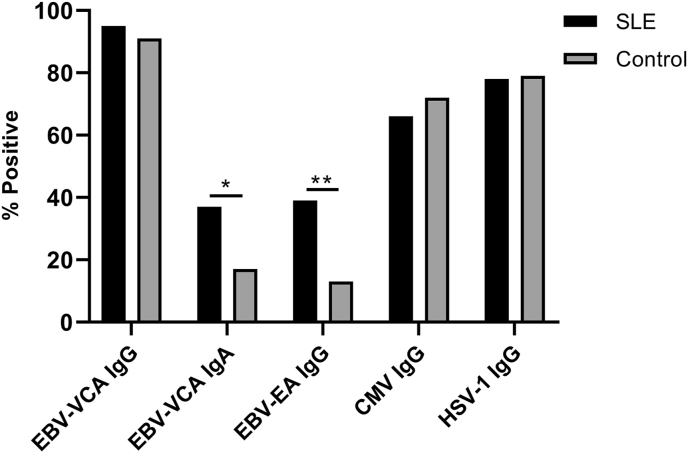
Systemic lupus erythematosus (SLE) patients exhibit more frequent EBV reactivation compared with controls. Seropositivity for EBV-viral capsid antigen (VCA) IgG and IgA, EBV-early antigen (EA) IgG, CMV-IgG, and HSV-1 IgG were determined by ELISA for SLE patients (n = 175) and controls (n = 47). *p < 0.05, **p < 0.01 by two-tailed z-score test of population proportions.

**Fig. 2:**
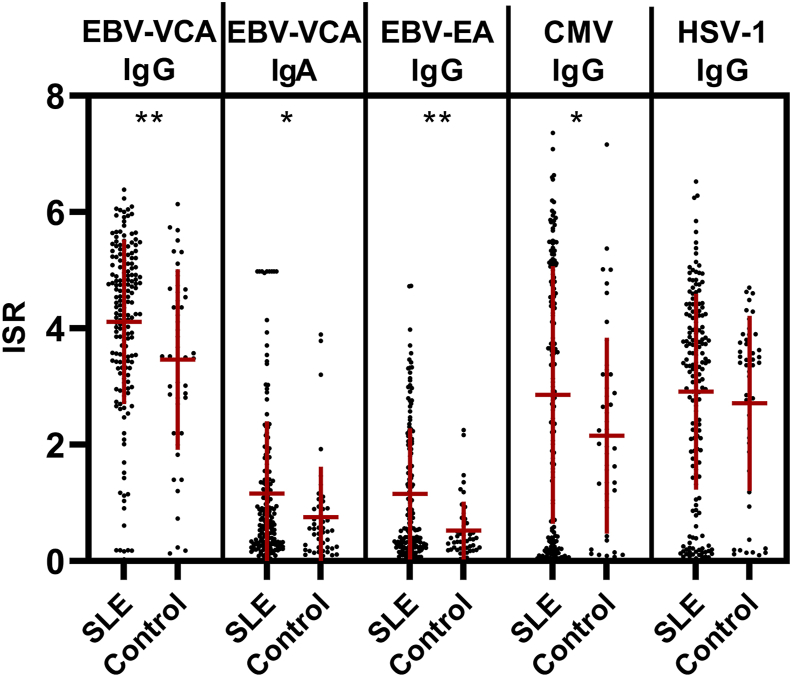
Systemic lupus erythematosus (SLE) patients exhibit higher levels of serological markers of Epstein-Barr virus (EBV) reactivation compared with controls. EBV-viral capsid antigen (VCA) IgG and IgA, EBV-early antigen (EA) IgG, cytomegalovirus (CMV) IgG, and herpes simplex virus (HSV-1) IgG antibody levels (international standardized ratio; ISR) were determined by ELISA for SLE patients (n = 175) and controls (n = 47). Each dot represents an independent sample, and data are represented as mean ± SD. *p < 0.05, **p < 0.01 by two-tailed student's t-test.

We first measured EBV-VCA IgG seropositivity to confirm previous EBV exposure among the study participants. Consistent with previous population studies, we found a similar near-ubiquitous exposure to EBV in SLE patients and healthy controls (95% vs 91%; p = 0.60) (Fig. 1). To determine if SLE patients experience more frequent EBV reactivation, we determined the seroprevalence and concentrations of EBV-EA IgG and EBV-VCA IgA, as well as concentrations of EBV-EA IgG. Compared to controls, significantly more SLE patients were seropositive for EBV-VCA IgA (37% vs 17%; p = 0.018) and EBV-EA IgG (39% vs 13%; p = 0.0011) (Fig. 1). SLE patients and controls exhibited similar seroprevalence of two other common herpesviruses, CMV (66% vs 72%; p = 0.49) and HSV-1 (78% vs 79%; p = 1.0) (Fig. 1). In addition, SLE patients exhibited significantly higher levels of EBV-VCA IgG, (mean ISR 4.1 vs 3.5; p = 0.0060), EBV-VCA IgA (mean ISR 1.2 vs 0.8; p = 0.022), EBV-EA IgG (mean ISR 1.2 vs 0.5; p = 0.0014), and CMV IgG (mean ISR 2.9 vs 2.2: p = 0.049) (Fig. 2). In contrast, SLE patients and controls had similar concentrations of HSV-1 IgG (Fig. 2). Collectively, serological analysis suggests that EBV reactivation is more common in SLE patients compared to controls and that they have higher humoral responses to EBV.

### 3.3 Elevated EBV reactivation is associated with higher disease activity in SLE patients

The functional consequences of EBV reactivation in SLE remain unclear [35]. To determine whether EBV reactivation is associated with more severe SLE disease activity, we measured antibody responses to EBV antigens in SLE patients experiencing high or low disease activity. As expected, SLE patients exhibited significantly higher antibody responses to EBV-EA IgG compared to controls, irrespective of disease activity (Table 2). Interestingly, SLE patients with high, but not low, disease activity exhibited significantly higher EBV-VCA IgG and EBV-VCA IgA levels compared to controls. Consistent with previous studies implicating EBV reactivation in SLE disease flare [36], SLE patients with high disease activity had higher EBV-VCA IgA (mean ISR 1.34 vs. 0.97; p = 0.041) and EBV-EA IgG levels (mean ISR 1.38 vs. 0.90; p = 0.007) compared to those with low disease activity (Table 2). However, EBV-VCA IgG levels did not significantly differ between SLE patients with high or low disease activity (Table 2). IgG responses to CMV and HSV-1 antigens also did not vary depending on SLE disease activity (Table 2), suggesting that the association is unique to EBV.

**Table 2:** Elevated concentrations of EBV-VCA (IgG and IgA) and EBV-EA (IgG) are associated with high SLE disease activity.

Serological measures, mean ISR (IQR)	SLE High (SLEDAI ≥6) n = 93	SLE Low (SLEDAI <4) n = 82	Control n = 47	SLE High vs. Control, p-value	SLE Low vs. Control, p-value	SLE High vs SLE Low, p-value
EBV-VCA IgG	4.24 (3.54, 5.21)	3.97 (3.11, 5.08)	3.46 (2.75, 4.64)	**0.005**	0.074	0.23
EBV-VCA IgA	1.34 (0.35, 1.75)	0.97 (0.30, 1.22)	0.75 (0.22, 0.90)	**0.004**	0.22	**0.041**
EBV-EA IgG	1.38 (0.33, 2.23)	0.90 (0.23, 1.32)	0.52 (0.21, 0.63)	**<0.001**	**0.004**	**0.007**
CMV IgG	3.04 (0.44, 4.93)	2.65 (0.21, 4.81)	2.15 (0.98, 3.10)	**0.013**	0.15	0.26
HSV-1 IgG	2.98 (1.91, 4.18)	2.83 (1.34, 4.22)	2.71 (1.71, 3.78)	0.35	0.70	0.55

Statistically significant values (p < 0.05) are in bold. Statistical significance was determined using a resampled student's t-test with n = 1000 permutations. CMV, cytomegalovirus; EA, early antigen; EBV, Epstein-Barr virus; HSV, herpes simplex virus; ISR, international standardized ratio; SLE, systemic lupus erythematosus; SLEDAI, systemic lupus erythematosus disease activity index; VCA, viral capsid antigen.

**Fig. 3:**
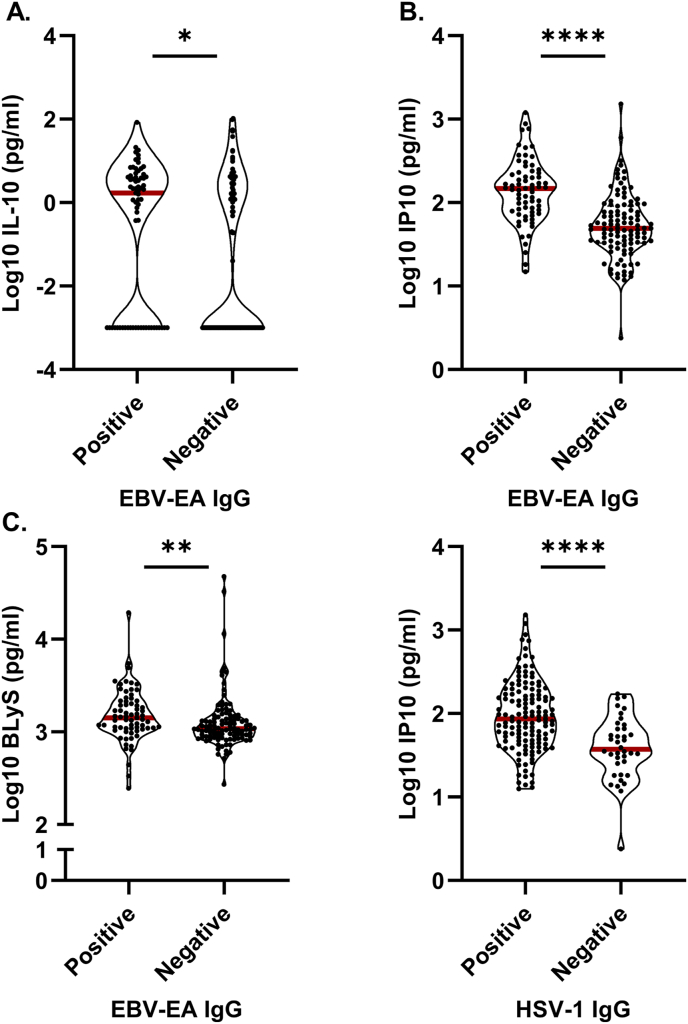
Seropositivity for Epstein-Barr virus (EBV)-early antigen (EA) IgG is associated with increased levels of EBV-related cytokine expression and type I IFN activity in systemic lupus erythematosus (SLE) patients. SLE patients were stratified based on seropositivity for (A–C) EBV-EA IgG (seropositive, n = 69; seronegative, n = 106) and (D) HSV-1 IgG (seropositive, n = 137; seronegative, n = 38). Plasma levels of (A) IL-10 and (B, D) IFN-induced protein 10 (IP-10) were determined using xMAP multiplex assays. Plasma levels of (C) B lymphocyte stimulator (BLyS) were determined by ELISA. Each dot represents an independent sample. *p < 0.05, **p < 0.01, ***p < 0.001, ****p < 0.0001 by two-tailed student's t-test.

To further explore the association between EBV reactivation and SLE disease activity, we stratified SLE patients based on EBV-EA IgG seropositivity and analyzed whether EBV reactivation correlated with elevated EBV-related cytokine expression and type I IFN activity. SLE patients who were EBV-EA IgG seropositive had significantly elevated levels of IL-10 (p = 0.0011), IP-10 (p < 0.001), and BLyS (p < 0.001) compared to those who were EBV-EA IgG negative (Fig. 3A–C). Consistent with the classical IFN response to viral infection [37], IP-10 was also elevated in SLE patients who were positive for HSV-1 IgG compared with those who were negative (p < 0.001) (Fig. 3D). Our findings suggest that EBV reactivation in SLE patients is associated with an increase in inflammatory and IFN-related responses.

### 3.4 EBV reactivation in SLE patients is associated with distinct phenotypic clusters

**Fig. 4:**
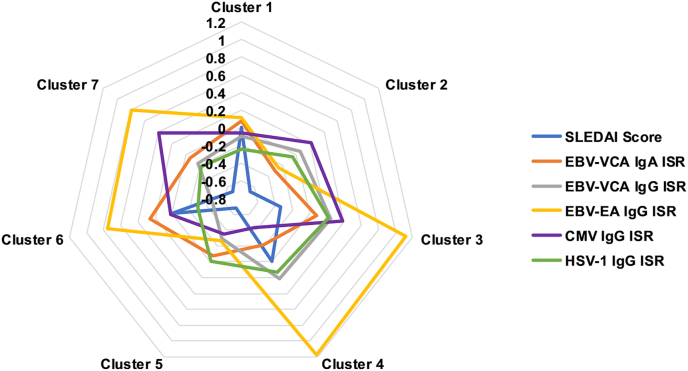
Epstein-Barr virus (EBV)-early antigen (EA) IgG responses are enriched in previously defined systemic lupus erythematosus (SLE) patient clusters. SLE patients were stratified based on molecularly defined SLE disease clusters [31]. Radar plots show SLEDAI scores and EBV-VCA IgG and IgA, EBV-EA IgG, CMV, and HSV-1 antibody levels in each patient cluster.

We previously defined molecular disease clusters for these SLE samples based on transcriptional module scores, soluble mediators, and lupus autoantibodies [31]. To determine if EBV reactivation and higher SLE disease activity corresponded to one or more SLE disease clusters, we used random forest analysis to group the SLE patients based on viral antibody titers and SLEDAI scores. Interestingly, EBV-EA IgG responses were enriched in clusters 3 and 4 (Fig. 4), which have higher expression of IFN and inflammatory or lymphoid and monocyte responses, respectively, and are associated with major organ involvement, including renal disease [31].

## 4 Discussion

In this study, we provide serologic evidence demonstrating that EBV reactivation not only correlates with the presence of SLE but also high disease activity and elevated levels of the soluble mediators IP-10, IL-10, and BLyS. Moreover, we found that SLE patients with EBV reactivation stratify into SLE phenotypic clusters with elevated IFN and inflammatory or lymphoid and monocyte responses. To our knowledge, this is the first study to show that EBV reactivation may influence SLE pathogenesis by promoting IFN and inflammatory immune pathways.

Consistent with previous studies [[15], [16], [17], [18]], we found that EBV reactivation is more prevalent in SLE patients compared to healthy controls. Although the exact triggers for EBV reactivation and why it occurs at a higher rate in SLE are not understood, SLE patients exhibit defective EBV-specific T cell cytokine secretion and cytotoxicity to both lytic and latent EBV antigens [[38], [39], [40], [41]]. As cell-mediated immunity is essential in maintaining EBV latency and preventing reactivation [42], this suggests one mechanism by which EBV reactivation may occur more frequently in SLE patients. PD-1 is upregulated on EBV-specific T cells in SLE patients, demonstrating T cell exhaustion, possibly resulting from frequent EBV reactivation [40]. In addition, SLE patients exhibit impaired cytokine responses to EBV antigens, which is correlated with high disease activity [41]. Alternatively, reactivation may occur due to B cell abnormalities in SLE patients [43]. Therefore, genetic factors predisposing to SLE may influence the host immune system, making it more susceptible to EBV reactivation.

We found that serologic markers of EBV reactivation are associated with elevated levels of IP-10 and are enriched in molecularly defined patient clusters with high IFN and inflammatory responses. Together, this suggests that EBV reactivation may contribute to IFN-associated immune pathways; however, the mechanistic pathways are still unknown. In vitro, EBV directly induces IFNα production by pDCs through the engagement of toll-like receptors 7 and 9 [29,30]. In addition, the latent viral protein EBNA-1 shares significant homology with lupus autoantigens, and immunization with these cross-reactive peptides promotes autoantibody production, inducing lupus-like autoimmunity in animal models [22,23,25,26,44,45]. The EBNA-1 epitope, PPPGRRP, cross-reacts with the PPPGMRPP sequence of the common SLE autoantigen Sm and is only expressed transiently [45,46]; therefore, EBV may specifically induce humoral autoimmunity following reactivation. Consistent with this hypothesis, serological measures of EBV reactivation correlate with autoantibody production in SLE patients [19,21,27,28]. Furthermore, EBV encodes vIL-10, which impairs apoptotic cell clearance by monocytes [24]. Together, excessive cell debris and autoantibodies form immune complexes, which also induce pDC IFNα production [[47], [48], [49]]. Collectively, these studies suggest that frequent EBV reactivation may exacerbate SLE disease activity by promoting IFN-associated inflammatory responses directly or indirectly through the generation of autoantibodies and impaired apoptotic clearance. However, further studies are required to test these hypotheses.

In summary, we propose that EBV reactivation exacerbates SLE pathogenesis, possibly through IFN-associated immune pathways. Current treatments for SLE include glucocorticoids and immunosuppressants, which have potent side-effects and are not effective in all patients due to the extensive heterogeneity of SLE. Therefore, precision medicine initiatives targeting specific subsets of SLE patients with unique genotypic and phenotypic characteristics are necessary [50]. We propose that stratifying patients based on serological markers of EBV reactivation would be beneficial for targeted therapies, as patients with high disease activity and rates of EBV reactivation may benefit from treatments that prevent EBV reactivation and spread [51].

## Funding

This work was supported in part by grants from the National Institutes of Health: U19AI082714, U01AI101934, UM1AI114292, U54GM104938, P30AR053483, and R01AR072401. The contents are solely the responsibility of the authors and do not necessarily represent the official views of the NIH.

## Credit author statement

**RAW:** Investigation, data curation, writing – review and editing. **LG:** Investigation, data curation, writing – review and editing. **ET:** Investigation, data curation, writing – review and editing. **CJG:** Investigation, supervision, writing – original draft, writing – review and editing. **JMK:** Data curation, formal analysis, visualization, writing – review and editing. **RLB:** Writing – original draft, writing – review and editing. **CAW:** Writing – original draft, writing – review and editing. **HC:** Data curation, formal analysis, visualization, writing – review and editing. **WD:** Data curation, writing – review and editing. **SRM:** Investigation, supervision, writing – review and editing. **SK:** Investigation, writing – review and editing. **RL:** Data curation, formal analysis, visualization, writing – review and editing. **CA:** Investigation, data curation, writing – review and editing. **EFC:** Investigation, data curation, writing – review and editing. **AT:** Investigation, data curation, writing – review and editing. **JTM:** Conceptualization, funding acquisition, formal analysis, investigation, resources, supervision, writing – review and editing. **JMG:** Conceptualization, funding acquisition, formal analysis, investigation, resources, supervision, writing – review and editing. **JAJ:** Conceptualization, funding acquisition, formal analysis, investigation, resources, supervision, writing – original draft, writing – review and editing.

## Declaration of competing interest

The authors declare the following financial interests/personal relationships which may be considered as potential competing interests: Cristina Arriens reports a relationship with Exagen Diagnostics Inc that includes: funding grants. Joan T Merrill reports a relationship with Exagen Diagnostics Inc that includes: funding grants. Joan T Merrill reports a relationship with GSK that includes: funding grants. Joan T Merrill reports a relationship with 10.13039/100008021Bristol Myers Squibb that includes: funding grants. Joan T Merrill reports a relationship with EMD Serono Inc that includes: consulting or advisory. Joan T Merrill reports a relationship with Eli Lilly and Company that includes: consulting or advisory. Joan T Merrill reports a relationship with RemeGen that includes: consulting or advisory. Joan T Merrill reports a relationship with GSK that includes: consulting or advisory. Joan T Merrill reports a relationship with UCB that includes: consulting or advisory. Joan T Merrill reports a relationship with Bristol Myers Squibb that includes: consulting or advisory. Joan T Merrill reports a relationship with AbbVie Inc that includes: consulting or advisory. Joan T Merrill reports a relationship with Amgen Inc that includes: consulting or advisory. Joan T Merrill reports a relationship with Daitchi Sankyo that includes: consulting or advisory. Joan T Merrill reports a relationship with Astellas Pharma US Inc that includes: consulting or advisory. Joan T Merrill reports a relationship with Pfizer that includes: consulting or advisory. Joan T Merrill reports a relationship with Genentech that includes: consulting or advisory. Joan T Merrill reports a relationship with AstraZeneca Pharmaceuticals LP that includes: consulting or advisory. Joan T Merrill reports a relationship with Jannsen that includes: consulting or advisory. Joan T Merrill reports a relationship with Servier that includes: consulting or advisory. Joan T Merrill reports a relationship with ILTOO Pharma that includes: consulting or advisory. Joan T Merrill reports a relationship with Xencor Inc that includes: consulting or advisory. Judith A. James reports a relationship with Progentec Diagnostics, Inc. that includes: funding grants. Judith A James reports a relationship with GSK that includes: consulting or advisory. Judith A James reports a relationship with Novartis that includes: consulting or advisory. Judith A James reports a relationship with Janssen Inc that includes: consulting or advisory.
